# Epidemiological Analysis of Traumatic Compartment Syndromes in Germany

**DOI:** 10.3390/jcm13061678

**Published:** 2024-03-14

**Authors:** Philipp Herrmann, Annette Eidmann, Felix F. Hochberger, Tizian Heinz, Dominik Rak, Manuel Weißenberger, Maximilian Rudert, Ioannis Stratos

**Affiliations:** Department of Orthopaedic Surgery, Koenig-Ludwig-Haus, Julius-Maximilians University Wuerzburg, Brettreichstrasse 11, 97074 Wuerzburg, Germanyi-stratos.klh@uni-wuerzburg.de (I.S.)

**Keywords:** traumatic compartment syndrome, demographics, Germany, billing data, epidemiological trends, healthcare data analysis

## Abstract

**Background**: Traumatic compartment syndrome is a critical condition that can lead to severe, lifelong disability. **Methods**: This retrospective study analyzed hospital billing data from 2015 to 2022, provided by the Federal Statistical Office of Germany, to examine the demographics and trends of traumatic compartment syndrome in Germany. The analysis included cases coded with ICD-10 codes T79.60 to T79.69 and any therapeutic OPS code starting with 5–79, focusing on diagnosis year, gender, ICD-10 code, and patient age. **Results**: The results showed that out of 13,305 cases, the majority were in the lower leg (44.4%), with males having a significantly higher incidence than females (2.3:1 ratio). A bimodal age distribution was observed, with peaks at 22–23 and 55 years. A notable annual decline of 43.87 cases in compartment syndrome was observed, with significant decreases across different genders and age groups, particularly in males under 40 (23.68 cases per year) and in the “foot” and “lower leg” categories (16.67 and 32.87 cases per year, respectively). **Conclusions**: The study highlights a declining trend in traumatic CS cases in Germany, with distinct demographic patterns. Through these findings, hospitals can adjust their therapeutic regimens, and it could increase awareness among healthcare professionals about this disease.

## 1. Introduction

Compartment syndrome (CS) is a severe and potentially limb-threatening medical condition, most commonly arising following traumatic injuries. The causes of CS can be divided into traumatic and non-traumatic. Epidemiological studies have well established that fractures are the most common cause of traumatic compartment syndromes, accounting for about 69–75% of all cases [1,2,3]. Nevertheless, traumatic CS can also be triggered by other diverse factors, including soft tissue injuries, vascular injuries, penetrating trauma, or severe thermal burns [4,5,6].

Nontraumatic causes of CS occur less frequently but can be attributed to a broad spectrum of conditions. Disturbances in blood coagulation, resulting from anticoagulation therapy or coagulation disorders, have been identified as contributors to non-traumatic CS [7]. Furthermore, nephrotic syndrome, revascularization procedures or treatments, or Group A streptococcus infections of muscles are non-traumatic causes of CS [8,9,10]. The pathophysiology of CS is characterized by increased pressure within a closed anatomical space, known as a compartment, leading to compromised blood flow and tissue perfusion. The resulting osmotic imbalance exacerbates intracellular edema, ultimately leading to necrosis. Consequently, this triggers the release of inflammatory substances in the affected area, further increasing tissue swelling and pressure within the compartment [11]. Early diagnosis and rapid surgical decompression are essential to prevent irreversible damage. It is well established that the treatment of CS is a fasciotomy of all involved compartments [12]. Adjunctive treatments include removing all constrictive dressings, elevating the affected limb, maintaining normotensive blood pressure, and providing oxygen supplementation. The diagnosis of CS and the decision for surgical intervention remain challenging. The primary element in diagnosing CS continues to be clinical assessment. An initial indication of CS often involves experiencing pain that is out of proportion and a heightened need for analgesics [13]. As the condition progresses, neurovascular symptoms arise due to prolonged ischemia, manifesting as pallor, pulselessness, paralysis, and paresthesia [14]. In cases where a compartment syndrome is clinically suspected, particularly in unconscious patients where diagnosis proves challenging, measuring intercompartmental pressure serves as a valuable tool to confirm the diagnosis.

Compartment syndromes may occur as a chronic or acute syndrome. The acute compartment syndrome is a surgical emergency with potentially devastating consequences. Untreated acute CS can result in necrosis, infection, paralysis, and limb amputation [15]. A recent study showed that only 69.2% of patients returned to their initial work [16]. This highlights the socioeconomic impact of CS and the significance of early diagnosis and accurate treatment. Numerous studies in the literature focus on the diagnosis and management of CS, with a particular emphasis on isolated body regions.

To date, there is a lack of literature detailing the prevalence of traumatic CS. Furthermore, research has not been conducted to identify which anatomical regions are most affected by CS. Moreover, a significant gap exists in the literature concerning comprehensive analyses encompassing trends and demographic characteristics associated with traumatic CS with simultaneous osteosyntheses in Germany. Our research aimed to fill this gap by examining cases of traumatic compartment syndrome that were treated concurrently with osteosynthetic procedures. The objective of our study was to investigate the demographics and overall trends in Germany.

## 2. Materials and Methods

### 2.1. Data Source and Data Structure

The Hospital billing data for patients with fracture- or luxation-associated compartment syndromes, who simultaneously received osteosynthetic treatment, were obtained from the Federal Statistical Office of Germany for the years 2015 to 2022. For the purpose of this study, we defined traumatic compartment syndrome as a coded compartment syndrome with a simultaneous coded osteosynthesis upon fracture or dislocation during the same hospital stay. The data included the number of patients diagnosed with a compartment syndrome and those undergoing reposition of a fracture or dislocation with osteosynthesis during the same hospital stay. To identify these patients, billing cases were searched for a diagnosis code (International Statistical Classification of Diseases and Related Health Problems-10 code) from T79.60 to T79.69 (indicating any traumatic compartment syndrome) combined with any therapeutic code (Operation and Procedure Classification System code) that starts with 5–79 (representing any open or closed reposition of a fracture or luxation using implants). The OPS Code is a classification system used in Germany to code medical procedures and interventions. The data output was provided to our group by an employee of the Federal Statistical Office of Germany following specific instructions. It included the year of diagnosis (2015–2022), gender (male and female), the ICD-10 code (T79.60 to T79.69), and patient age (grouped in 5-year intervals). The data were presented in a grouped format and can be downloaded from the URL https://github.com/ioannis-stratos/compartment (generated and accessed on 7 January 2024).

### 2.2. Data Processing

This dataset was provided in a wide format. For further analysis, it was transformed from a wide format to a long format using R (version 2023.12.0, R-Studio; Boston, MA, USA) and the ‘reshape2’ library. Subgroup analysis was performed using the ICD-10 codes T79.60 (compartment syndrome of the upper extremities; “upper extremity group”), T79.61 (compartment syndrome of the hip and thigh; “hip and thigh group”), T79.62 (compartment syndrome of the lower leg; “lower leg group”), T79.63 (compartment syndrome of the foot; “foot group”), and T79.68 and T79.69 (compartment syndrome of other or unspecified localizations; “unspecified group”). Further subgroup analysis included gender, year of diagnosis, and patient age (grouped as “≥40 years” and “<40 years”). These subgroups were calculated and analyzed in tables using Tableau Desktop (version 2023.3, Tableau Software, Seattle, WA, USA).

### 2.3. Statistical Analysis

Using GraphPad Prism (version 10.1.1; GraphPad Software; San Diego, CA, USA), linear regression analyses were performed and diagrams generated. Linear regression analysis is a statistical method used to understand the relationship between a dependent variable and one or more independent variables. The F-test was used to determine if the overall significance of the linear regression model was significantly different from zero. For nonlinear regression analysis, Lorentzian distributions, a continuous probability distribution, were employed. The χ^2^-test was applied to assess differences in group distributions. The significance level was set at *p* ≤ 0.05 for the statistical analyses.

## 3. Results

**Figure 1:**
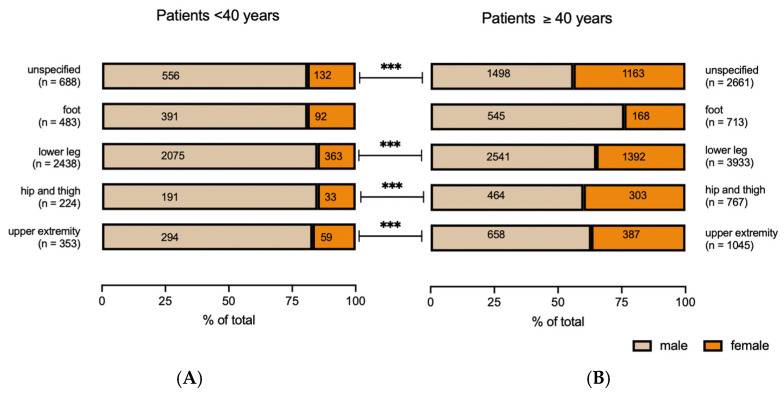
Number and % of total of compartment syndromes categorized by localization (foot, upper extremity, hip and thigh, lower leg, or unspecified location) and age of patients ((**A**): <40 years; (**B**): ≥40 years). χ^2^-test; *** *p* < 0.001.

During the period from 2015 to 2022, we reviewed a total of 13,305 cases of traumatic compartment syndromes. Of these, 61% manifested in the lower extremities and 11% in the upper extremities. A significant portion of cases lacked precise localization classification (25.2%). Most traumatic compartment syndromes occurred in males (4092 cases in females vs. 9213 in males), resulting in a female to male ratio 1:2.3 (Table 1). Subgroup analysis, differentiating between younger patients (<40 years) and older patients (≥40 years), revealed that most younger patients were male (male to female ratio of 5.1:1). In contrast, the older age group showed an increased proportion of females (male to female ratio of 1.7:1) (Figure 1). The distribution of males and females within younger and older patients was equal in the group “foot”. For all other localizations (unspecified, lower leg, hip and thigh, upper extremities), the male to female ratio decreased significantly in patients above 40 years of age (Figure 1).

**Figure 2:**
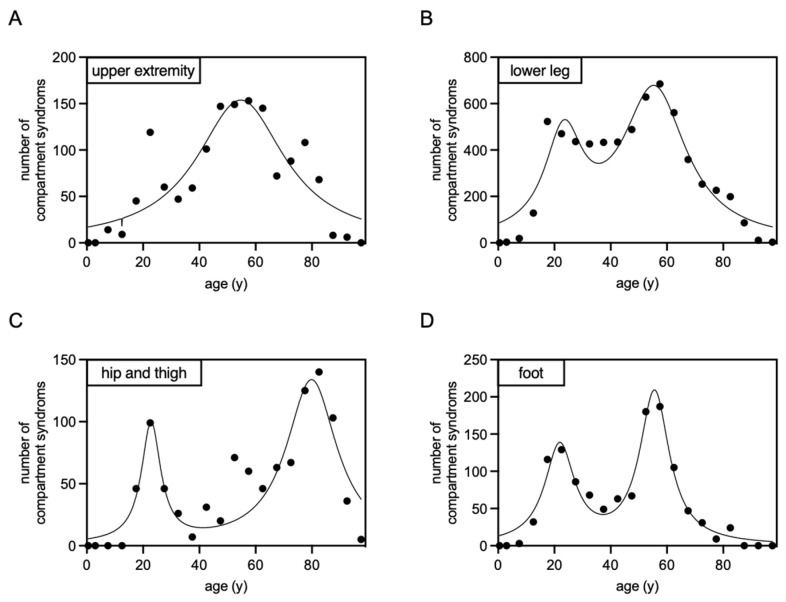
Non-linear regression analysis, employing Lorentzian distributions, was conducted on compartment syndrome cases following osteosynthesis between 2015 and 2022, categorized by the affected area ((**A**): upper extremity; (**B**): lower leg; (**C**): hip and thigh; (**D**): foot). The resulting graphs display the frequency of compartment syndromes in correlation with the patients’ ages.

In most localizations, the age distribution shows a bimodal trend. The initial peak occurs at the age of 22 and 23, with distinct peaks at different sites: the “foot” at 21.88 years, the “lower leg” at 23.32 years, and the “hip and thigh” at 22.84 years. The second peak is observed at 55 years, with the “foot” peaking at 55.58 years and the “lower leg” at 55.49 years. However, for the “hip and thigh,” the second peak is considerably later, at 79.88 years. By contrast, the age distribution for the upper extremity is unimodal, reaching its peak at 54.72 years for the “upper extremity” (Figure 2).

**Figure 3:**
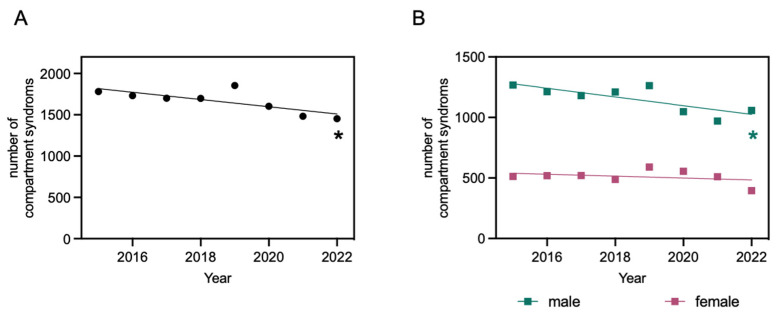
Compartment syndromes after osteosynthesis from 2015 to 2022. Linear regression function: y = −43.87x + 90,213; R^2^ = 0.5842; * significantly non-zero slope of the line, evidenced by an F-statistic of 8.429 and a *p*-value of 0.03 (**A**). Compartment syndromes after osteosynthesis from 2015 to 2022, categorized by patients’ sex (male and female). Linear regression function for males: y = −36.04x + 73,890; R^2^ = 0.6325; * significantly non-zero slope of the line, evidenced by an F-statistic of 10.33 and *p* = 0.02. Linear regression function for females: y = −7.83x + 16,323; R^2^ = 0.1147; slope of the line not significantly different from zero, evidenced by an F-statistic of 0.7774 and *p* = 0.41 (**B**).

**Figure 4:**
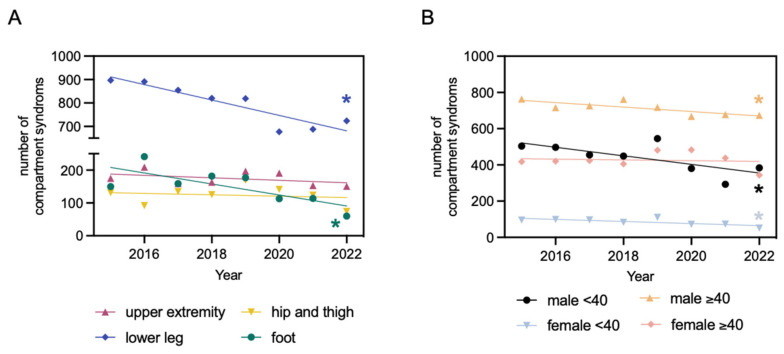
Compartment syndromes after osteosynthesis from 2015 to 2022, categorized by localization (foot, upper extremity, hip and thigh, lower leg). Linear regression function for the foot: y = −16.76x + 33,983; R^2^ = 0.5652; * significantly non-zero slope of the line, evidenced by an F-statistic of 7.8 and *p* = 0.03. Linear regression function for the upper extremity: y = −3.786x + 7816; R^2^ = 0.1794; slope of the line not significantly different from zero, evidenced by an F-statistic of 1.312 and *p* = 0.3. Linear regression function for the hip and thigh: y = −2.107x + 4377; R^2^ = 0.03103; slope of the line not significantly different from zero, evidenced by an F-statistic of 0.1922 and *p* = 0.68. Linear regression function for the lower leg: y = −32.87x + 67,143; R^2^ = 0.8277; * significantly non-zero slope of the line, evidenced by an F-statistic of 28.83 and *p* = 0.002 (**A**). Compartment syndromes after osteosynthesis from 2015 to 2022, categorized by patients age (<40 years and ≥40 years) and sex (male and female). Linear regression function for males under 40: y = −23.68x + 48,234; R^2^ = 0.501; * significantly non-zero slope of the line, evidenced by an F-statistic of 6.025 and *p* = 0.05. Linear regression function for males over 40: y = −12.36x + 25,656; R^2^ = 0.6429; * significantly non-zero slope of the line, evidenced by an F-statistic of 10.8 and *p* = 0.02. Linear regression function for females under 40: y = −5.750x + 11,691; R^2^ = 0.5522; * significantly non-zero slope of the line, evidenced by an F-statistic of 7.398 and *p* = 0.03. Linear regression function for females over 40: y = −2.083x + 4632; R^2^ = 0.01332; slope of the line not significantly different from zero, evidenced by an F-statistic of 0.08101 and *p* = 0.79 (**B**).

Throughout the analyzed period, all traumatic compartment syndromes exhibited a significant decline in cases per year, amounting to a reduction of 43.87 cases annually (Figure 3). A classification based on the sex also shows a significant decline of 36.04 cases per year for males and a not significant decline of 7.83 cases per year for females (Figure 3). Upon further classification based on localization, distinct patterns emerged. For the localizations “foot” and “lower leg”, a statistically significant decrease in cases per year was identified (“foot”: 16.67 fewer cases per year; “lower leg”: 32.87 fewer cases per year). For “upper extremity” and “hip and thigh”, there was also a decline in cases per year observed, but it was statistically not significant (“upper extremity”: 3.78 fewer cases per year; “hip and thigh”: 2.10 fewer cases per year) (Figure 4).

The subgroup analysis further confirms a yearly decrease in the incidence of compartment syndrome following osteosynthesis. Notably, a significant yearly decrease was observed in all male patient groups, with a reduction of 23.68 cases in males under 40 and 12.36 cases in males over 40. Among females under 40, the data also indicate a significant yearly decrease, with 5.75 fewer cases. However, for females over 40, the yearly reduction was not statistically significant, showing only a modest decrease of 2.08 cases (Figure 4).

## 4. Discussion

Our study uncovered clear trends and demographic features regarding compartment syndrome (CS) following trauma. Predominantly, cases were observed in the lower leg (44.4%), with a notable proportion occurring in the upper extremities (10.5%). These data are consistent with the literature. Notably, McQueen et al. demonstrated that fractures are the primary cause of CS at a rate of 69%, with the most prevalent location being the diaphysis of the tibia (36%), followed by the distal radius as the second most common site [15].

Our research examined 13,305 cases of traumatic compartment syndromes that were treated concurrently with osteosynthetic procedures, recorded in Germany from 2015 to 2022. Data from the German Statistical Office indicate that the country’s average population during this period was approximately 83,053,327 [17]. This translates to an incidence rate of 2 per 100,000 individuals for traumatic compartment syndrome. With this incidence, the compartment syndrome following trauma represents a rare clinical entity that needs more attention.

A significant gender-based difference was observed, revealing a higher incidence of traumatic compartment syndromes in males compared to females, resulting in a female-to-male ratio of approximately 1:2.3 across all anatomical locations, including the upper extremity, thigh, lower leg, and foot. One plausible explanation for this observation is the greater likelihood of men being involved in high-energy accidents, often resulting in diverse bone fractures [18]. Additionally, McQueen et al. proposed that young men possess a larger proportion of muscle, while the fascial shell does not proportionally expand, potentially contributing to the observed patterns. Conversely, older men exhibit reduced muscular tissue, allowing more space for muscle swelling following injury [15]. Most studies indicate that youth is one of the strongest predictors for developing CS [19,20].

In our investigation, a bimodal pattern in the age distribution was observed for most anatomical locations, with initial peaks at 22 to 23 years, followed by a second peak at 55 years of age. Interestingly, the upper extremity displayed a unimodal pattern, peaking at 55 years. Despite indications in the dataset of a bimodal age distribution curve for the upper extremity group, its statistical significance was not confirmed across different statistical models. A plausible explanation for the prominence of the 55-year age group could be the occurrence of a midlife crisis, often experienced between ages 40 and 60. In this context, some individuals may engage in riskier activities or sports like motorcycling. Studies have shown that older victims of motorcycle crashes typically present with more severe injuries than their younger counterparts [21,22].

Furthermore, the period from 1955 to 1969 in Germany corresponds to the baby boomer generation [23]. Shoob et al. demonstrated that Baby Boomers significantly impacted the absolute numbers of hospitalizations for coronary heart disease and stroke in 2000 compared to individuals aged 45–54 in 1990 and 1980 [24]. This demographic phenomenon could contribute to an increased incidence of compartment syndrome within this specific age group. Interestingly, the second peak in the age distribution for “hip and thigh” was considerably later at 79.88 years. This could be explained by the increased incidence of femoral fractures in elderly patients combined with an oral anticoagulation therapy [25].

We observed a temporal declining trend in the incidence of compartment syndrome in our study, despite the increasing incidence of fractures in Germany from 2009 to 2019 [25]. The decline in traumatic compartment syndrome could be attributed to several factors. Advancements in surgical techniques and technologies, including osteosynthesis, have led to more precise and less invasive surgeries, potentially reducing the likelihood of complications like compartment syndrome. Enhanced imaging modalities, improved fixation devices, and better preoperative planning might collectively contribute to a decrease in postoperative complications.

Increased awareness among healthcare professionals about the risk factors and early signs of compartment syndrome could also play a crucial role in the observed decline of CS. Emphasis on medical education to monitor patients for symptoms of CS leads to earlier detection and intervention. As noted, the diagnosis of CS and the decision for surgical intervention remain challenging, evidenced by a study highlighting diverse rates of diagnosis and therapeutic interventions for CS [26]. Improving educational initiatives, raising awareness of CS, and focused sensitization may have resulted in a reduction in the indications for hasty or even unnecessary fasciotomies.

Recent diagnostic tools for identifying CS, such as continuous intracompartmental pressure monitoring, have demonstrated high sensitivity and specificity and should be considered for all high-risk patients [27]. This may contribute to further declining numbers. Moreover, the cautious approach to diagnosing compartment syndrome might also be a factor. Additionally, advances in rehabilitation strategies and postoperative care, focusing on minimizing swelling and optimizing tissue healing, may contribute to the reduction in the incidence of compartment syndromes. It is essential to consider these factors and their collective influence on the observed decline.

While many investigations have focused on individuals treated at a single trauma center, our study utilized a dataset from the Federal Statistical Office of Germany, covering 2015 to 2022. These data span diagnoses, treatments, and patient demographics, collected from numerous healthcare facilities across the nation. This comprehensive dataset allows for a thorough examination of demographic trends across Germany. Utilizing internationally recognized coding systems like the International Classification of Diseases (ICD) for diagnoses and the German procedure classification system (OPS) for treatments, the data maintain a high level of consistency. This standardization is crucial and allows a comparison with other datasets. However, the data source is not without its limitations. One of the primary concerns is the potential for coding errors and inconsistencies. Despite the standardization, data entry mistakes or variations in interpreting coding guidelines are possible. Additionally, since the data are tailored more for administrative and financial purposes, it often lacks detailed clinical information. In our dataset, approximately a quarter of cases lacked precise localization classification, highlighting one of our concerns. It is also possible that different hospitals and healthcare providers might employ varying practices in coding and data entry, which could introduce biases into the dataset. Additionally, our study included only traumatic compartment syndromes combined with simultaneously osteosynthetic treatment. Other traumatic causes such as spontaneous bleeding into a compartment, injection of pressurized fluid into the compartment, or a too-tight cast and all non-traumatic compartment syndromes were excluded [28,29]. Initially, we aimed to include all patients with traumatic compartment syndrome. Unfortunately, the billing data provided by the Statistical Office of Germany were not specific enough to answer this question, because non-traumatic compartment syndromes (e.g., after revascularization) were included in the dataset. To overcome this analysis bias, we had to refine our search query and search for the coded diagnosis of compartment syndrome in combination with any osteosynthesis that was performed during the same hospital stay. By doing so, we had the advantage of excluding non-traumatic compartment syndromes from our analysis but, on the other hand, faced the disadvantage of not being able to identify isolated traumatic compartment syndromes that did not require osteosynthesis. This approach made our results more accurate and specific to our research question.

Our current study provides a snapshot of compartment syndromes in Germany, offering insights within specific temporal and regional boundaries. The reasons behind the observed decrease in the number of compartment syndrome cases warrant further exploration in subsequent clinical studies. To this end, both prospective and retrospective clinical investigations are of great significance, as they can offer a deeper understanding of the underlying factors contributing to this trend.

## Figures and Tables

**Table 1:** Summary of total numbers of traumatic compartment syndromes between 2015 and 2022, categorized by localization (foot, upper extremity, hip and thigh, lower leg, and unspecific location) and patient sex (male and female).

	Foot	Upper Extremity	Hip and Thigh	Lower Leg	Unspecified Location	Total
Male patients (% of total)	936 10.2%	952 10.3%	655 7.1%	4616 50.1%	2054 22.3%	9213 100%
Female patients (% of total)	260 6.4%	446 10.9%	336 8.2%	1755 42.9%	1295 31.6%	4092 100%
Sum male & female (% of total)	1196 9.0%	1398 10.5%	991 7.4%	5911 44.4%	3349 25.2%	13,305 100%
Ratio male:female	3.6	2.1	1.9	2.6	1.6	2.3

## Data Availability

All source data can be downloaded from the URL https://github.com/ioannis-stratos/compartment (generated and accessed on 7 January 2024).
